# The “Raspberry” Vaccination Scar, and Its Guarantee of Protection from Small Pox

**Published:** 1882-12

**Authors:** 


### THE "RASPBERRY" VACCINATION SCAR, AND ITS GUARANTEE OF PROTECTION FROM SMALL-POX.

Dr. Richard C. Newtown, Assistant Surgeon, U. S. Army, Fort Cummings, New Mexico, writes: "I have read with interest during the past summer a good deal of matter relative to vaccination and vaccine virus, which *The Record* and other jouruals have set before their readers. And although the gentlemen who are interested in vaccine farms are thoroughly convinced that their particular brand of virus is all sufficient, others have grave doubts as to what form of vaccine it is best to use. Without in any manner considering myself competent to enter into this discussion, I wish to relate, very briefly, a case of my own, because, as it seems to me, it throws some light upon the matter, and may be of help to both practitioners and patients.

"I have always used fresh bovine virus and have generally been satisfied with the results. I even supposed that the 'raspberry' scar indicated that a modified protection against small-pox had been obtained.

"I noticed that a writer in *The Record* denounced this peculiar scar as of no avail because it follows a worthless bovine (?) vaccination.

"Last August I saw J. R.——, aged twenty-eight; American; healthy; good family history; by occupation a railway employee, although he was then in a mining camp. At first he had the lumbar pains, fever, cough, and great depression; these soon developed into a well-marked case of discrete smallpox. The man was isolated, and the best care possible, under adverse circumstances, was taken of him. He did not recollect having been vaccinated in childhood. He had nursed a case of smallpox; after which, he had been vaccinated; this was about five weeks before I saw him. He said that a doctor whose name he did not know had vaccinated a large number of railroad men, himself among them, and as nearly as I could judge from the patient's account, the physician had used a cone of bovine virus. The man said that the vaccination had "taken" well, and he supposed himself amply protected. This conversation occurred about ten days after I first saw the case. I rather doubted the word of my patient, who thereupon pulled up his sleeve and showed me a distinct 'raspberry' scar on his arm, and this was surrounded by the umbilicated pustules of small-pox. No other vaccination marks could be discovered on either arm, although of course, some might have been present and not discernible.

"The man died in the third week of the disease, after the case had passed out of my hands. I heard, however, that he ran out in a violent shower of rain. With better surroundings I should have expected a favorable result; although I no longer believe in the modified protective influence of a vaccination leaving a 'raspberry' scar."—*Med. Record.*

